# Development and Psychometric Evaluation of the Antibiotic Knowledge and Consumption Tool (AKCT)

**DOI:** 10.3390/antibiotics11121744

**Published:** 2022-12-02

**Authors:** Sanah Hasan, Hana Sulieman, Husam Babi, Samir Bloukh

**Affiliations:** 1 Department of Clinical Sciences, College of Pharmacy and Health Sciences, Ajman University, Ajman P.O. Box 346, United Arab Emirates; 2 Center of Medical and Bio-Allied Health Sciences, Ajman University, Ajman P.O. Box 346, United Arab Emirates; 3 Department of Mathematics and Statistics, American University of Sharjah, Sharjah P.O. Box 26666, United Arab Emirates

**Keywords:** antibiotic knowledge, tool development, translation and cultural adaptation, Arabic, United Arab Emirates

## Abstract

Knowledge of antibiotics and awareness of microbial resistance are essential for appropriate antibiotic consumption. This study aimed to develop and validate a measure of antibiotic knowledge and consumption (AKCT) and to make it available in the Arabic language and context. The tool was developed and applied on individuals ≥ 18 years, with mastery of Arabic or English. Exploratory factor analysis using principal-component analysis tested the psychometric properties of the items. AKCT scores were compared with the Infectious Numeracy Test (INT) scores to establish convergent validity. Cronbach’s α > 0.7 measured reliability. Three hundred-eighty-six participants completed the questionnaire, achieving a 95.3% response rate. Five components were retained after factor analysis: Side-effects and resistance, Access to antibiotics, Recovery after use, Antibiotics use indications, and Body response. Cronbach’s α = 0.85. The mean ± SD of AKCT = 9.82 ± 3.85 (range = 7–20); lowest scores were related to “Side-effects and resistance” (2.32 ± 2.00, max = 7) and “Antibiotic use indications” (1.61 ± 1.29, max = 5). Scores on the AKCT and INT positively correlated. The AKCT is a valuable, valid, and reliable tool developed for measurement of antibiotic knowledge and consumption behaviors to identify specific areas needing improvements; hence, targeted interventions are devised.

## 1. Introduction

Antibiotic resistance is a growing problem in the management of infectious diseases that leads to real threats on public health [1,2]. Antibiotic misuse and unnecessary use are major contributors to the development of microbial resistance [3,4] and are common in both developed and developing countries [4,5,6,7]. This takes various shapes and forms including overuse of antibiotics, improper use, failure to complete treatment, skipping of doses, and re-use of leftover medicines [4,8,9]. Frequently, misconceptions about antibiotics lead to their inappropriate use in upper-respiratory-tract viral infections and suboptimal use such as shortening the course of treatment [10]. Inadequate knowledge about antibiotics and lack of awareness of resistance are commonly reported as the main reasons for the inappropriate use of antibiotics.

It has been postulated that the lack of knowledge about antibiotics and awareness of antibiotic resistance are thought to influence patient and parent demand for antibiotic prescribing [11]. Frequently, patients mandate, or physicians perceive patients to expect, a prescription for an antibiotic even when it is clearly not indicated; this has been reported to have a large influence on physician prescribing [4,12]. Similarly, the practice of self-medication with antibiotics is widespread within populations, especially in the developing world [13], and is largely influenced by the lack of knowledge and misconceptions regarding antimicrobials by the general public [7,12].

Enhancing public knowledge about antibiotics and resistance by the use of educational interventions has strongly been advocated [14]. Education is also believed to be a vital component of any intervention planned to improve prescribing practices related to antibiotics [15]. There are widespread differences in antibiotic use among various populations and these are related to the level of knowledge of antibiotics and awareness of antimicrobial resistance [16]; hence, it is crucial to assess this knowledge before any interventional programs are designed as they need to be tailored to the needs of that particular population. A number of studies related to antibiotic knowledge have globally been conducted [17,18,19,20,21]. These studies consistently found that the public generally had a suboptimal but variable level of knowledge about antibiotics, which could limit the application of universal interventions.

Overuse of antibiotics is common in Arabic-speaking countries; self-medication rates ranged from 32–42% in Lebanon [22] to 32–62% in Jordan [23]. Antibiotics in self-medication are mainly used for treatment of upper-respiratory-tract symptoms, urinary tract infections, or gastrointestinal symptoms, and were encouraged to be consumed because of their availability at home as leftovers from previous use, usually as a recommendation from family and/or friends [24]. Another pattern of antibiotic misuse in Arabic-speaking countries is largely of not completing the full course of treatment, which ranged from 29% to 86% [25,26].

Knowledge and beliefs are social cognitive factors at an individual level that influence health-related behavior, including the behavior of consuming antibiotics. Knowledge by itself is not enough to change behavior but does play an important role in influencing beliefs and attitudes in relation to a particular behavior [27]. Consequently, in the context of antibiotic use, inappropriate knowledge of antibiotics potentially leads to misconceptions that could negatively influence their consumption. Therefore, assessing the level of knowledge related to antibiotics could help identify those who have potential for inappropriate consumption behaviors that increase the risk of bacterial resistance. This will consequently help in developing targeted intervention and education of individuals based on their level of knowledge and type of antibiotic consumption behaviors. Currently, no validated tools are available to measure the knowledge of antibiotics and resistance and the associated antibiotic consumption behaviors displayed by populations. Similarly, in the Arabic-speaking world, these tools do not exist. Therefore, this study aimed at developing and validating a measure of knowledge of antibiotics and resistance, and consumption behaviors of individuals in the general population and to make this measure available in the Arabic language and context.

## 2. Results

A total number of 405 potential participants who met the inclusion criteria were approached, and 386 completed the questionnaire, achieving a response rate of 95.3%. The participants spent an average of 10–15 min to complete the questionnaire.

### 2.1. Participant Characteristics

Most participants were female, 263 (68.1%); aged between 18 and 30 years, 250 (65%); Arab, 352 (91.2%); had at least university/college education, 208 (53.9%). They were also considered to have low income, 272 (70.5%); did not have a persistent or long-lasting illness, 348 (90.2%) (Table 1). Clearly, participants were not familiar with antibiotics, 278 (72%); could not give specific names of antibiotics; out of three antibiotic names from a total of six medications, they scored a Mean ± SD of 1.47 ± 0.77. Many participants used antibiotics without obtaining a prescription, 111 (28.8%); more than one third, 134 (34.7%), used them at least twice; while 76 (19.7%) used them more than 5 times in the past year (Table 1). Participant responses on questionnaire items revealed that most participants, 261 (67.62%), knew that antibiotics were used for bacterial infections, while 183 (47.4%) believed antibiotics were used to treat viral infections, another 188 (48.7%) believed antibiotics were used for the common cold, and only 95 (24.6%) believed that “the more antibiotics we used in society, the higher the risk that resistance develops” (Table 2).

### 2.2. Structural Validity—Exploratory Factor Analysis

Factor analysis revealed an unforced five-factor structure, with eigenvalues greater than one, which explained 52.5% of the variance. Item 12 had poor loading (<0.3) on any of the factors; hence, it was decided to remove it from the scale, while item 5 had a loading of 0.392 on one of the factors and correlated well with other items within the factor; hence, it was retained. The factor structure was clear, as could be seen in the component matrix (Table 3): Factor 1: Side-effects and resistance, α = 0.856; Factor 2: Access to antibiotics, α = 0.792; Factor 3: Recovery after use, α = 0.592; Factor 4: Antibiotics use indications, α = 0.631; Factor 5: Body response, α = 0.504 (Table 3). All the items had factor loadings higher than 0.40 (except for item 5 as explained before), varying from 0.392 in item 5 to 0.797 in item 23. Each item was aggregated to the component on which it presented the highest loading value. The Kaiser–Meyer–Olkin measure of sampling adequacy was 0.8216 and Bartlett’s Test of Sphericity was highly significant (χ2 = 2521.078, df = 22, *p <* 0.001), demonstrating that the factor analysis was adequate to the data.

### 2.3. Internal Consistency Reliability

The reliability analysis showed good internal consistency for the 23-item scale, with a Cronbach’s internal consistency coefficient of 0.848. Furthermore, the five factors also showed good internal consistency; some had a Cronbach’s internal consistency coefficient < 0.7 (Table 3), which is still considered satisfactory for short tests (factors) [28].

The mean score of the AKCT was 9.82 ± 3.85 (range = 7–20, max. = 20); lowest scores were related to the Side-effects and resistance factor (Mean ± SD of 2.32 ± 2.00, max = 7) and Antibiotic use indications factor (Mean ± SD = 1.61 (1.29), max = 5) (Table 4). Results of the multivariate analysis of variance showed that gender had no effect on any of the knowledge or consumption factors; however, older participants (≥51 years) had higher knowledge of Antibiotic uses and indications (2.23 ± 1.33, *p* = 0.016), while those with higher postgraduate education presented with higher levels of knowledge concerning Side-effects of medications and resistance (2.03 ± 1.91, *p* = 0.001), Recovery after use (2.03 ± 1.91, *p* = 0.046), and Body response (1.68 ± 0.89, *p* = 0.000). Likewise, participants with higher income (>30,000 AED) presented higher scores of knowledge than those with a lower level as related to Antibiotic use and indications (1.96 ± 1.08, *p* = 0.043), Side-effects and resistance (3.67 ± 1.97, *p* = 0.008), and Body response (1.95 ± 0.80, *p* = 0.024) (Table 4). Comparison of antibiotic knowledge scores based on the participant frequency of use of antibiotics revealed that those who never used antibiotics in the past 12 months scored the lowest knowledge scores (Mean ± SD = 8.69 ± 4.58), and lower scores were also achieved by those who used antibiotics “2–5 times” (Mean ± SD = 10.0 ± 4.1). Knowledge scores in both those who used antibiotics “once” (Mean ± SD = 10.64 ± 4.0) and “2–5 times” (Mean ± SD = 11.1 ± 3.6) gave comparably and significantly higher mean scores than those who “never” or used antibiotics “more than 5” times, *p* = 0.008.

### 2.4. Convergent Validity

Higher knowledge scores as measured by the AKCT were significantly correlated with the scores of the INT (r = 0.205, *p <* 0.01); similarly, factor 1 within the AKCT “Side-effects and resistance” was significantly correlated with the average scores of the INT (r = 0.162, *p* = 0.05), although the level of correlation was weak (Table 5).

## 3. Discussion

A new measure of antibiotic knowledge and consumption scale has been developed and psychometrically tested, and is readily available for use in the clinic, community pharmacy, and the outpatient hospital setting. The tool may be used to assess the level of knowledge and consumption behavior of individuals and, hence, targeted intervention could be designed to enhance knowledge, modify demands on physician prescribing, and optimize consumption practices related to antibiotics.

Our findings suggest that the participants had poor knowledge of antibiotics reflected by the low performance on naming antibiotics and/or identifying antibiotic names and by their low scores on the AKCT, which is consistent in several studies exploring population knowledge of antibiotics [17,18,19,20,21,29]. Although most participants knew that antibiotics were used for bacterial infections, only less than half recognized that they may not be used for viral infections. Additionally, participants achieved lower scores in the Antibiotic use indications factor. Practically, this meant that measuring knowledge of antibiotics and their use should go beyond the obvious general use for bacterial infections, and that details of what is a bacterial or viral infection are not so obvious for most participants. The term “germs” commonly used in lay language communications [30] could possibly be contributing to the existing confusion related to the difference between bacteria and viruses. Hence, there may be benefit in specifically using the terms ‘bacteria’ and ‘virus’ when it comes to explaining infections and the associated prescribing decisions to the patient [7].

Participants also specifically performed low on the Side-effects and resistance factor among other factors with only (43.52%) recognizing that “Bacteria can become resistant to antibiotics”, and (37.82%) knowing that “Antibiotics often cause side effects such as diarrhea”. Additionally, only (24.61%) of participants identified that “The more antibiotics we used in society, the higher the risk that resistance develops”, which is particularly lower than what was reported from China (54.36%) [29], Malaysia (59.1%) [31], and Korea (70.1%) [9], and possibly explains the careless pattern of antibiotic use in a large group of participants in this study and highlights the crucial need for patient education in this area.

People’s misconceptions of antibiotics could possibly lead to inappropriate antibiotic consumption including misuse and self-medication [10]. In a review about antibiotic use in developing countries, the belief was that an antibiotic was “an extraordinary” or “a powerful” or “a magical” medicine, which was able to prevent and cure any disease [32]. This view of antibiotics among members of the population leads them either to demand a prescription from the physician for antibiotics even in a minor self-limiting illness, or to self-medicate with antibiotics. Physicians find themselves required to satisfy patient expectations and provide the prescription despite the lack of clinical need [30]. Paradoxically, parents with low knowledge scores were found more likely to expect an antibiotic during a clinic visit for their child’s respiratory illness, and were more likely to report receiving an antibiotic for a diagnosis that was considered as nonbacterial [30]. Irrational antibiotic prescribing has also been known to contribute to irrational patient use; in one study, rational prescribing of antibiotics was reported in only 42.9% of cases [33], which showed that not only patients but also physicians were contributing to irrational antibiotic use. This is of great importance as previous prescribing of antibiotics influenced patient expectations of antibiotics during subsequent visits. In addition to the internet, leftover stock, across country supply, and veterinary sources, community pharmacies remain the main source of nonprescription antibiotic supply [34,35,36,37,38]. As healthcare professionals are the main source of antibiotic information to patients [19], physicians and pharmacists play an important role in shaping public knowledge of antibiotics, possibly leading to an improvement in patients’ behavior related to antibiotic consumption. Both physicians and pharmacists should be inclined to engage in more rational prescribing and dispensing of antibiotics and provide more information about their appropriate use; frequently, patients complained of the scarcity of the information they received from healthcare providers about antibiotics [19].

It has been reported that the pressure for antibiotic prescribing by patients could start as early as the beginning of the clinic visit and could continue throughout [39]. Therefore, agenda mapping [40] has been proposed where communication at the start of the visit included the agenda of both the physician and the patient, and emphasis on taking time to elicit the problems and concerns of the patient was undertaken. This approach led to higher patient satisfaction, reducing late arising concerns, and improving understanding, time management, treatment adherence, and health outcomes. Findings showed that general practitioners who made room for the story of patients by using active listening techniques received more information about the ideas, concerns, and expectations (ICEs) and were able to successfully reassure patients that there was no reason to give antibiotics [41].

Findings in this study confirmed that age, level of participant education, and socioeconomic class were associated with the knowledge of antibiotics scores [7,17,19,30,31,38]. However, the knowledge of antibiotic scores in our study was low in general, indicating that education and literacy do not necessarily lead to more knowledge about antibiotics, and that interventions to enhance knowledge and positive consumption behaviors still need to target educated people and those belonging to a higher socioeconomic class. Our study also showed that the lowest knowledge scores were associated with no use, or very frequent use (overuse), of antibiotics. Clearly, some of the participants who lacked knowledge did not attempt to use antibiotics, while others extensively used them. This resonates with other studies citing that individuals with poor knowledge of antibiotics tended to overestimate their knowledge and this overestimation and overconfidence in self-knowledge enhanced the prevalence of self-medication behavior [19].

The findings also show an association between the AKCT scores and the infectious numeracy scores represented by the INT, which was expected. The little published literature of numeracy and knowledge in infectious diseases showed limited numeracy skills to be associated with lower knowledge of the condition [42], which meant that assessment of patients’ knowledge could indicate the level of numeracy. Similarly, numeracy and knowledge in other diseases have been found to be correlated [43,44,45]. More specifically in this study, numeracy was found to be associated with knowledge of Side-effects and resistance of antibiotics; therefore, interventional education aiming at improving the knowledge of antibiotics and side-effects and resistance may also be helpful in improving numeracy skills needed while consuming antibiotics.

In the Arabic-speaking world, there has been no validated tool to measure the level of knowledge about antibiotics and the type of consumption behaviors that these populations are engaged in. This tool is available now, and it is simple and easy to use in the clinic, community pharmacy, or the outpatient setting before an antibiotic is considered for prescribing or dispensing. The tool should also be instrumental in defining specific areas of patient knowledge and consumption that require targeted intervention and education.

## 4. Materials and Methods

### 4.1. Tool Development

**Figure 1:**
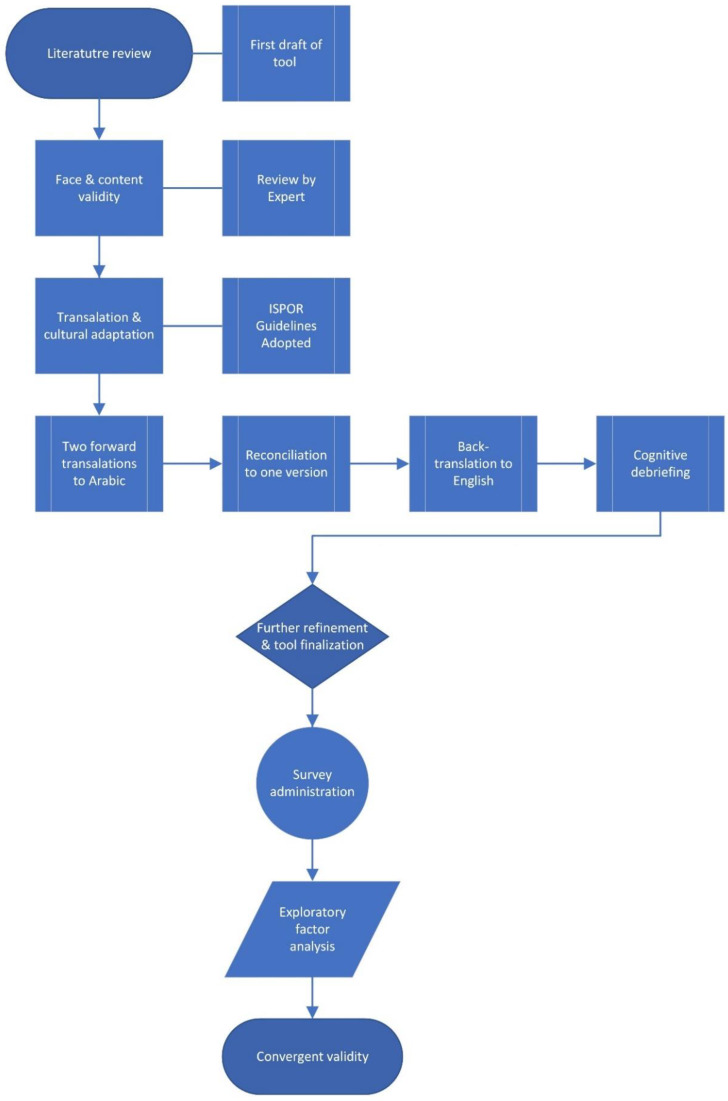
Tool development and validation.

Figure 1 shows a flowchart of the process utilized in developing and validating the tool. In constructing the tool, both the international [7,46] and local [8] literature was consulted. As the questionnaire was set to assess the knowledge and consumption behavior of antibiotics, the research team hypothesized items covering basic background knowledge about antibiotics, side-effects, resistance, and use patterns to be included [7,29].

The first part of the questionnaire collected information related to the participants such as: sociodemographics of gender, age, country of origin, education level, monthly income, the presence of chronic disease, and any previous medical education or training. Other questions included participant familiarity with antibiotics through their ability to name and/or recognize antibiotic names, if they ever used antibiotics without a prescription, and the number of times they had used antibiotics in the past 12 months.

The second part of the questionnaire comprised 24 questions assessing participant background knowledge about antibiotics (e.g., “Antibiotics can be used to treat bacterial infections”, “You can use antibiotics when you have a common cold”, and “If one feels better, he/she should stop using the antibiotic as soon as feeling better”); side-effects (e.g., “Antibiotics often cause side effects such as diarrhea”); resistance (e.g., “The more antibiotics we used in society, the higher the risk that resistance develops” and “Resistance to antibiotics could spread from one country to another when people travel back and forth between countries”); use patterns (e.g., “I usually obtain antibiotics from a pharmacy without a doctor’s visit”, “In my home, leftover antibiotics can be saved for personal future use or given to someone else”, and “Sometimes I buy antibiotics online, without having to see a doctor”) [7,8,46]. Participants answered the 24 questions on a “Yes”, “No”, and “I don’t know” (“not applicable”) scale. The “I don’t know” (“not applicable”) option was added to decrease participant guessing, and to indicate to the participant that it would not be expected that they answered all questions correctly. The correct responses/appropriate (safe) behaviors related to antibiotic use were summed up to give the total Antibiotic Knowledge and Consumption Tool (AKCT) score with higher scores representing a higher level of participant knowledge about antibiotics and engagement in more appropriate/safe consumption behavior.

### 4.2. Face and Content Validity

A first draft of the tool was produced by one of the authors (HB) based on a literature review as previously described. Content validity of the tool was assessed by a panel of four experts, including one academic professor in the field of Clinical Pharmacy/Pharmacy Practice, an epidemiologist, a practicing pharmacist, and a microbiologist. The panel met on several occasions to discuss the appropriateness of items, completeness of the questionnaire, and clarity of wording. Additionally, four research assistants with a background in pharmacy practice attended the meetings and contributed to the discussions of item selection and wording. Each item had to be approved by all members of the panel; in cases where there was disagreement concerning an item, it was then refined until a consensus was reached.

### 4.3. Tool Translation to Arabic

The ISPOR guidelines for translation and cultural adaptation of patient-reported outcomes [47] were followed to translate and culturally adapt the English version to the Arabic context. The developed questionnaire was forward-translated to Arabic by two independent professional translators, which led to producing two Arabic translations of the tool; the two translations were reconciled by the research panel to produce one version. A third translator (an academic) translated the reconciled Arabic version back to English. This new English version was compared to the original tool to resolve any differences. All members on the research team were bilingual of Arabic and English; hence, they worked together to revise all versions of the instrument by checking meanings and refining minor differences.

### 4.4. Cognitive Debriefings

Cognitive interviews were conducted by the research assistants on a sample of 10 participants from various socioeconomic and education backgrounds. The cognitive interviews were conducted at the participant’s workplace to eliminate any source of bias or inconvenience for them. Participants gave feedback to the research assistants regarding the choice, clarity, and completeness of items. The time needed to complete the questionnaire was also estimated. The cognitive interviews were part of establishing the face and content validity of the tool and helped to further refine and finalize it.

### 4.5. Inclusion and Exclusion Criteria

Those who were ≥18 years and proficient in Arabic or English were included in this study. Those who had mental illness or cognitive impairment were excluded.

### 4.6. Sample Size

The most recommended ratio of participants to items in tool development is 10–20 participants per item [48], so we opted for a ratio of 15 participants per item; hence, the sample size needed was 360 based on 24 questionnaire items. However, the calculated sample size was increased by approximately 8% to account for incomplete responses leading to a final suggested sample of 388 participants.

### 4.7. Survey Administration

The survey was distributed in paper-format to a convenience sample of participants visiting public places such as shopping malls and community pharmacies in three cities of Dubai, Sharjah, and Ajman in the United Arab Emirates. The four trained research assistants approached those who were seemingly above 18 years of age and screened them for inclusion in the study. Those who met the inclusion criteria were briefed about the study aims and what participating entailed, and were handed a more detailed explanatory information sheet. Subsequently, those agreeing to participate signed a consent form and filled out the questionnaire on the spot. All completed questionnaires were placed in a closed box and returned to the principal investigator for subsequent handling. The questionnaires were distributed by hand as this was seen, at the time, as the most efficient way to collect data in the absence of an effective postal system in the UAE. The survey was distributed between 1 December 2019 and 3 March 2020.

### 4.8. Convergent Validity

Numeracy and knowledge in other diseases have been found to be correlated [43,44,45]. Therefore, to establish convergent validity of the tool, the total scores achieved on the AKCT were compared to the Infectious Numeracy Test (INT) scores achieved by the participants. The INT was a 9-item scale developed to assess participant numeracy related to the use of antibiotics and was factored on two dimensions: “Mathematical knowledge and problem-solving” and “Numeracy-related practices and experience” [42]. As with higher achieved scores on the AKCT representing higher knowledge and more appropriate consumption behavior, higher scores on the INT represented higher numeracy skills.

### 4.9. Data Analysis

Descriptive statistics were calculated for participant demographics and other collected information, such as the level of obtained education, monthly income, previous medical education, presence of chronic disease, familiarity with antibiotic names, and consumption behaviors related to antibiotics.

The responses of the participants in each item of the AKCT were coded as 3 = “Correct”, 2 = “Incorrect”, and 1 = “I don’t know”. The total number of correct responses was calculated for each participant, with higher scores representing better knowledge of antibiotics and more appropriate consumption behaviors.

For the statistical analyses, Minitab Statistical Software Version 21 was used. A Cronbach alpha greater than 0.7 was used to measure the internal consistency of the scale. Exploratory factor analysis (EFA) was performed on the 24 items to determine the distinct areas of antibiotic knowledge covered by the questionnaire, using the principal-component analysis with varimax rotation. The Kaiser-criterion (eigenvalue > 1) and scree plot were examined to determine the maximum number of factors. An item was disregarded when the factor loading was lower than 0.4. The internal consistency of the resulting factors was assessed by the inter-item correlation matrix and Cronbach’s alpha (α).

Differences in each of the extracted factors among the sociodemographic variables of gender, age, education, and monthly income were tested using multivariate analyses of variance. A 5% significance level was used to declare a significant effect. Convergent validity was assessed through the Pearson correlation coefficient between the AKCT and INT scores, which were expected to be correlated.

## 5. Limitations

In this study, most participants in this study were young (between the age of 18 and 30, representing the young age average of the ex-patriate majority in the population in this country) women and 45% had a college degree, which could compromise the generalizability of the findings. The tool developed in this study should still apply to populations that will need it most: young women not only taking care of themselves but also of other family members including children. Convenience sampling could have led to selection bias, where those who may seem more literate and knowledgeable about antibiotics would elect to participate; however, our findings still showed a suboptimal level of knowledge of antibiotics in this population in general. Furthermore, plans are underway to apply the tool in more homogenous Arabic-speaking populations in Jordan, Palestine, and Egypt to further validate the tool and include a more diverse sample.

## 6. Conclusions

A measure of antibiotic knowledge and consumption behavior has been developed and psychometrically tested. The AKCT is available in English and Arabic for use internationally. Despite the educated participants and those belonging to a higher socioeconomic class achieving higher knowledge of antibiotics and consumption behavior scores, the overall scores in the AKCT were suboptimal. This demonstrated that low knowledge and misuse of antibiotics were prevalent across all segments of the population. The AKCT tool can identify the specific areas of low knowledge so targeted interventions and intensive education can be provided. AKCT scores correlated with numeracy scores associated with antibiotic use; therefore it may be supportive in identifying those with low numeracy, so key skills in antibiotic use are further reinforced.

## Figures and Tables

**Table 1:** Sociodemographics and antibiotic familiarity and consumption of participants, N = 386.

**Sociodemographic Information**	**N (%)**
Gender: Male Female	123 (31.9) 263 (68.1)
Age: 18–30 31–40 41–50 ≥51	250 (65.0) 64 (16.5) 42 (10.8) 30 (7.7)
Nationality: UAE National Other Arab Non-Arab	214 (55.5) 138 (35.7) 34 (8.8)
Education: Tertiary school or less Postgraduate Higher degree	178 (46.1) 175 (45.3) 33 (8.6)
Monthly Income (AED): <10,000 10,000–20,000 20,000–30,000 >30,000	272 (70.5) 57 (14.8) 33 (8.5) 24 (6.2)
Do you have any persistent or long-lasting illness? Yes No	38 (9.8) 348 (90.2)
Have you had any medical/health related education? Yes No	25 (6.5) 361 (93.5)
**Familiarity and consumption of antibiotics**	**N (%)**
Please name below some of the antibiotics you have heard of: Correct Incorrect	108 (28.0) 278 (72.0)
Have you ever used antibiotics without a doctor’s prescription? Yes No I don’t know	111 (28.8) 161 (41.7) 114 (29.5)
How many times have you consumed antibiotics during the past 12 months? Never Once only 2–5 times More than 5	35 (9.1) 141 (36.5) 134 (34.7) 76 (19.7)
Do you consume antibiotics when your body temperature is ________? More than 37 °C More than 37.5 °C More than 38 °C More than 38.5 °C I don’t use antibiotics unless prescribed by my physician	191 (49.5) 74 (19.2) 32 (8.3) 18 (4.6) 71 (18.4)
Which of the following six medications is an antibiotic? *(Correct response = 3)*	*Mean*±*St.D* 1.47 ± 0.77

**Table 2:** Participant responses to questionnaire items *.

Item	Correct/ Approp. N (%)	Incorrect/ In-Approp. N (%)	I Don’t Know N (%)
Antibiotics can be used to treat bacterial infections	**261 (67.62)**	**53 (13.73)**	**72 (18.62)**
Antibiotics can be used to treat viral infections	134 (34.72)	**183 (47.41)**	69 (17.88)
The body can fight mild infections on its own without antibiotics	**277 (71.76)**	62 (16.06)	47 (12.18)
You can use antibiotics when you have a common cold	109 (28.24)	**188 (48.70)**	89 (23.06)
You can use antibiotics when you have pneumonia (lung infection)	**160 (41.45)**	72 (18.65)	154 (39.90)
You should always use antibiotics if one’s mucous becomes colored when having a cold	108 (27.98)	**81 (20.98)**	197 (51.04)
You should always use antibiotics when you have sore throat	144 (37.13)	**132 (34.20)**	110 (28.50)
An ear infection in a 3–6 year old child has to be treated with antibiotics	102 (26.42)	**105 (27.20)**	179 (46.37)
If one feels better, he/she should stop using the antibiotic as soon as feeling better	153 (39.64)	**166 (43.01)**	67 (17.36)
Antibiotics are used to kill all bacteria in the body	137 (35.49)	**122 (31.61)**	127 (32.90)
Antibiotics make one recover faster when having a cold	122 (31.61)	**140 (36.27)**	124 (32.12)
I usually obtain antibiotics from a pharmacy without a doctor’s visit	177 (45.85)	**163 (42.23)**	46 (11.92)
In my home, leftover antibiotics can be saved for personal future use or given to someone else	183 (47.41)	**166 (43.01)**	37 (9.59)
Sometimes I am able to acquire antibiotics from relatives or acquaintances, without having to be examined by a doctor	280 (72.54)	**75 (19.43)**	31 (8.03)
Sometimes I buy antibiotics online, without having to see a doctor.	256 (66.32)	**108 (27.98)**	22 (5.07)
If I get an infection, I often wait and see. (i.e., rest and take it easy), and see if the infection goes away on its own	**291 (56.74)**	75 (19.43)	20 (5.18)
Antibiotics often cause side effects such as diarrhea	**146 (37.82)**	73 (18.91)	167 (43.26)
Antibiotics can cause negative effects on the body’s own bacteria	**144 (37.31)**	89 (23.06)	153 (39.64)
Bacteria can become resistant to antibiotics	**168 (43.52)**	43 (11.14)	175 (45.34)
Resistant bacteria can spread from one patient to another	**138 (35.75)**	93 (24.09)	155 (40.16)
The more antibiotics we used in society, the higher the risk that resistance develops	**95 (24.61)**	50 (12.95)	241 (62.44)
Antibiotic use in animals can have an effect on antibiotic treatment for humans	**106 (27.46)**	61 (15.80)	219 (56.46)
Resistance to antibiotics could spread from one country to another when people travel back and forth between countries	**100 (25.91)**	47 (12.18)	239 (61.92)

* Bolded items are considered correctly placed.

**Table 3:** Rotated component matrix and consistency reliability of the 23-item knowledge scale.

Item		Factor				
		1	2	3	4	5
1	Antibiotics can be used to treat bacterial infections	*	*	*	*	0.643
2	Antibiotics can be used to treat viral infections	*	*	*	*	0.788
3	The body can fight mild infections on its own without antibiotics	*	*	*	*	0.479
4	You can use antibiotics when you have a common cold	*	*	*	−0.433	*
5	You can use antibiotics when you have pneumonia (lung infection)	*	*	*	−0.392	*
6	You should always use antibiotics if mucous becomes colored when having a cold	*	*	*	−0.707	*
7	You should always use antibiotics when you have sore throat	*	*	*	−0.641	*
8	An ear infection in a 3–6 year old child has to be treated with antibiotics	*	*	*	−0.690	*
9	If one feels better, he/she should stop using the antibiotic as soon as feeling better	*	*	0.654	*	*
10	Antibiotics are used to kill all bacteria in the body	*	*	0.627	*	*
11	Antibiotics make one recover faster when having a cold	*	*	0.535	*	*
12	I usually obtain antibiotics from a pharmacy without a doctor’s visit	*	0.702	*	*	*
13	In my home, leftover antibiotics can be saved for personal future use or given to someone else	*	0.726	*	*	*
14	Sometimes I am able to acquire antibiotics from relatives or acquaintances, without having to be examined by a doctor	*	0.792	*	*	*
15	Sometimes I buy antibiotics online, without having to see a doctor.	*	0.795	*	*	*
16	If I get an infection, I often wait and see. (i.e., rest and take it easy), and see if the infection goes away on its own	*	0.637	*	*	*
17	Antibiotics often cause side effects such as diarrhea	0.657	*	*	*	*
18	Antibiotics can cause negative effects on the body’s own bacteria	0.697	*	*	*	*
19	Bacteria can become resistant to antibiotics	0.616	*	*	*	*
20	Resistant bacteria can spread from one patient to another	0.658	*	*	*	*
21	The more antibiotics we used in society, the higher the risk that resistance develops	0.781	*	*	*	*
22	Antibiotic use in animals can have an effect on antibiotic treatment for humans	0.791	*	*	*	*
23	Resistance to antibiotics could spread from one country to another when people travel back and forth between countries	0.797	*	*	*	*
**Cronbach’s Alpha**		0.856	0.792	0.592	0.631	0.504
**Cronbach’s Alpha for total knowledge scale**		0.848				

* Loadings < |0.40|.

**Table 4:** Comparisons of factor scores over demographic variables.

Variable	N	Factor 1 (7 Items) Mean ± St.D P 2.32 ± 2.00	Factor 2 (5 Items) Mean ± St.D P 3.08 ± 1.52	Factor 3 (3 Items) Mean ± St.D P 1.07 ± 0.98	Factor 4 (5 Items) Mean ± St.D P 1.61 ± 1.29	Factor 5 (3 Items) Mean ± St.D P 1.74 ± 0.92
**Gender** Male Female	123 263	2.39 ± 2.21 0.66 2.29 ± 1.90	2.93 ± 1.65 0.21 3.14 ± 1.46	0.95 ± 0.97 0.13 1.11 ± 0.98	1.61 ± 1.38 0.96 1.61 ± 1.25	1.74 ± 0.99 0.92 1.74 ± 0.89
**Age** 18–30 31–40 41–50 ≥51	250 64 42 30	2.27 ± 1.95 0.29 2.15 ± 1.84 2.40 ± 2.20 2.97 ± 2.37	3.18 ± 1.47 0.20 2.85 ± 1.65 2.74 ± 1.78 3.10 ± 1.24	0.98 ± 0.96 0.12 1.28 ± 0.92 1.19 ± 1.06 1.13 ± 1.04	1.63 ± 1.24 **0.016** 1.35 ± 1.34 1.43 ± 1.40 2.23 ± 1.33	1.71 ± 0.89 0.53 1.72 ± 1.03 1.78 ± 0.97 1.96 ± 0.89
**Education** Tertiary school or less Postgraduate Higher degree	178 175 33	2.03 ± 1.91 **0.001** 2.41 ± 2.02 3.39 ± 2.03	3.00 ± 1.57 0.70 3.12 ± 1.50 3.18 ± 1.44	2.03 ± 1.91 **0.046** 2.41 ± 2.02 3.39 ± 2.03	1.51 ± 1.33 0.30 1.67 ± 1.24 1.85 ± 1.35	1.68 ± 0.89 **0.000** 1.67 ± 0.93 2.42 ± 0.75
**Monthly Income** **<**10,000 10,000–20,000 20,000–30,000 **>**3000	272 57 33 24	2.17 ± 1.98 **0.008** 2.24 ± 1.83 2.36 ± 2.23 3.67 ± 1.97	2.98 ± 1.58 0.38 3.26 ± 1.40 3.24 ± 1.37 3.37 ± 1.31	1.02 ± 0.95 0.14 1.29 ± 1.00 0.91 ± 1.01 1.25 ± 1.07	1.49 ± 1.28 **0.043** 1.88 ± 1.40 1.90 ± 1.28 1.96 ± 1.08	1.68 ± 0.92 **0.024** 2.03 ± 0.90 1.57 ± 0.93 1.95 ± 0.80
**AKCT** Mean ± St.D = 9.82 ± 3.85	386	2.32 ± 2.00	3.08 ± 1.52	1.07 ± 0.98	1.61 ± 1.29	1.74 ± 0.92

**Table 5:** Pearson correlation coefficient between the AKCT and INT.

	INT (Total Score)
Antibiotic knowledge (total score)	0.205 **
Factor 1—Side-effects and resistance	0.162 *
Factor 2—Access to antibiotics	−0.060
Factor 3—Recovery after use	0.125
Factor 4—Antibiotics use indications	−0.025
Factor 5—Body response	−0.030

* *p* = 0.05; ** *p* < 0.01.

## Data Availability

The data presented in this study are available on request from the corresponding author. The data are not publicly available due to confidentiality concerns.
